# Comparison of NICE criteria with the BOADICEA multifactorial risk model to guide breast cancer risk assessment and referral amongst women under age 50 within primary care

**DOI:** 10.1038/s41416-026-03547-2

**Published:** 2026-08-04

**Authors:** Reuben Frost, Lorenzo Ficorella, Amy Berrington de Gonzalez, Antonis C. Antoniou, Montserrat García-Closas, Juliet A. Usher-Smith

**Affiliations:** 1 Division of Genetics and Epidemiology, The Institute of Cancer Research, London, UK; 2 Centre for Cancer Genetic Epidemiology, Department of Public Health and Primary Care, University of Cambridge, Cambridge, UK; 3 Cancer Epidemiology and Prevention Research Unit, The Institute of Cancer Research and Imperial College London, London, UK; 4 Primary Care Unit, Department of Public Health and Primary Care, University of Cambridge, Cambridge, UK

**Keywords:** Risk factors, Preventive medicine, Translational research

## Abstract

**Background:**

The National Institute for Health and Care Excellence (NICE) recommends that women at above-population-level risk of breast cancer (BC) are offered enhanced screening and risk-reducing interventions. Identification of these women largely relies on referral through primary care based on family history criteria.

**Methods:**

We compared the performance of the NICE criteria with versions of the BOADICEA multifactorial risk model in identifying women who developed BC within 10 years. Analyses included 1258 women aged under 50 years with family history, reproductive, lifestyle and polygenic risk data recruited to the Generations Study between 2004 and 2011 (392 BC cases), weighted to represent the UK population with respect to age and family history of BC.

**Results:**

NICE criteria identified 4.4% of women who developed BC within 10 years amongst 1.4% of women who met referral criteria. The full BOADICEA model identified up to 35% of women who developed BC, but with lower specificity and 26.5% of women meeting referral criteria.

**Conclusions:**

NICE family history-based criteria applied to women under 50 miss most BCs occurring within 10 years. Multifactorial risk assessment in primary care could substantially improve the identification of women at risk who could be offered interventions before being invited to population-based screening.

## Background

Breast cancer (BC) is the most common cancer worldwide, accounting for approximately 25% of cancers and 15% of cancer deaths in women [1]. In England, the National Institute for Health and Care Excellence (NICE) recommends that women at moderate- or high-risk of BC should be offered risk-reducing medications alongside enhanced breast imaging from age 30 to 50, depending on risk level [2]. Trials have shown that annual mammography between age 40 and 50 reduces mortality from BC by 12 to 29% [3], and risk-reducing medications can reduce the relative risk of BC in women without a personal history of BC by 30–60% if taken for 3–5 years [4, 5].

Women above population-level risk due to having a relative with a high-risk pathogenic genetic variant (e.g. in *BRCA1/2*) are often identified via cascade testing led by specialist genetics clinics. Identification of all other women relies on referral from primary care. In England, guidance from NICE recommends that healthcare professionals in primary care take a first- and second-degree family history when women present with concerns around their family history or, in some clinical circumstances, when women over 35 years are using an oral contraceptive pill or considering long-term hormone replacement therapy [2]. The criteria for referral are based on a combination of the number of relatives with cancer and the age and sex of those relatives. For those women meeting criteria for referral, formal BC risk assessment using risk prediction models is then recommended in secondary care or specialist genetics services.

Studies have consistently shown that the ability to identify women at moderate- or high-risk is maximised when the joint effects of genetic, demographic, behavioural and hormonal/reproductive risk factors are considered alongside family history in multifactorial BC risk prediction models [6–9]. Current referral criteria in primary care are, therefore, likely to miss a large number of those at above-population-level risk; it is estimated that only a small proportion (<10%) of those at moderate or high risk are currently identified through current practice [10].

While multifactorial BC risk prediction models are currently only recommended for use by NICE in secondary care or specialist genetics services, there is interest in implementing proactive BC risk assessment within primary care [11]. However, collecting additional risk factor information, in particular comprehensive first- and second-degree family history including both affected and unaffected relatives, is resource-intensive, both for women providing initial information and healthcare professionals conducting subsequent verification [12, 13]. There is, therefore, a need to balance this additional data collection burden with the gains in accuracy of risk assessment in this population.

No studies have directly compared the current NICE criteria based on family history with the use of multifactorial models within a primary care population of women under age 50. Additionally, the incremental value of individual risk factors, including data on unaffected relatives, within this population, is not known. The aim of this study is to compare the use of current NICE criteria with combinations of risk factors considered in the multifactorial Breast and Ovarian Analysis of Disease Incidence and Carrier Estimation Algorithm (BOADICEA) risk prediction model for identifying women under age 50 at above-population-level risk to inform potential risk assessment and referral pathways for women under age 50 and prior to eligibility for population-based screening programmes.

## Methods

### Study population

The Generations Study (GS) is a prospective cohort of women in the UK designed to investigate BC aetiology. Full details of the cohort are described elsewhere [14]. In brief, women aged over 16 years were recruited between 2004 and 2011. At study entry, women completed a baseline questionnaire on BC risk factors—including reproductive and menstrual history, exogenous hormone intake, exercise, benign breast disease, alcohol, smoking, height, weight, education and family history of cancer—and provided a blood sample. Detailed family history of cancer was self-reported at baseline, with any cancer history being requested for the participant’s parents, grandparents, siblings and children, and BC history for any blood relative. Genotyping data from blood DNA derived from genome-wide arrays in a nested case-control sample of study participants of White ethnicity were used to calculate the 313-single nucleotide polymorphism BC polygenic score (PGS) (PGS-313) [15]. Follow-up data on site-specific cancer incidence and cause-specific mortality were obtained through spontaneous reports and confirmed by linkage to the national cancer registry and mortality data [14]. Deaths, emigrations and other exits were also obtained through ‘flagging’ on the NHS Central Registers.

**Fig. 1:**
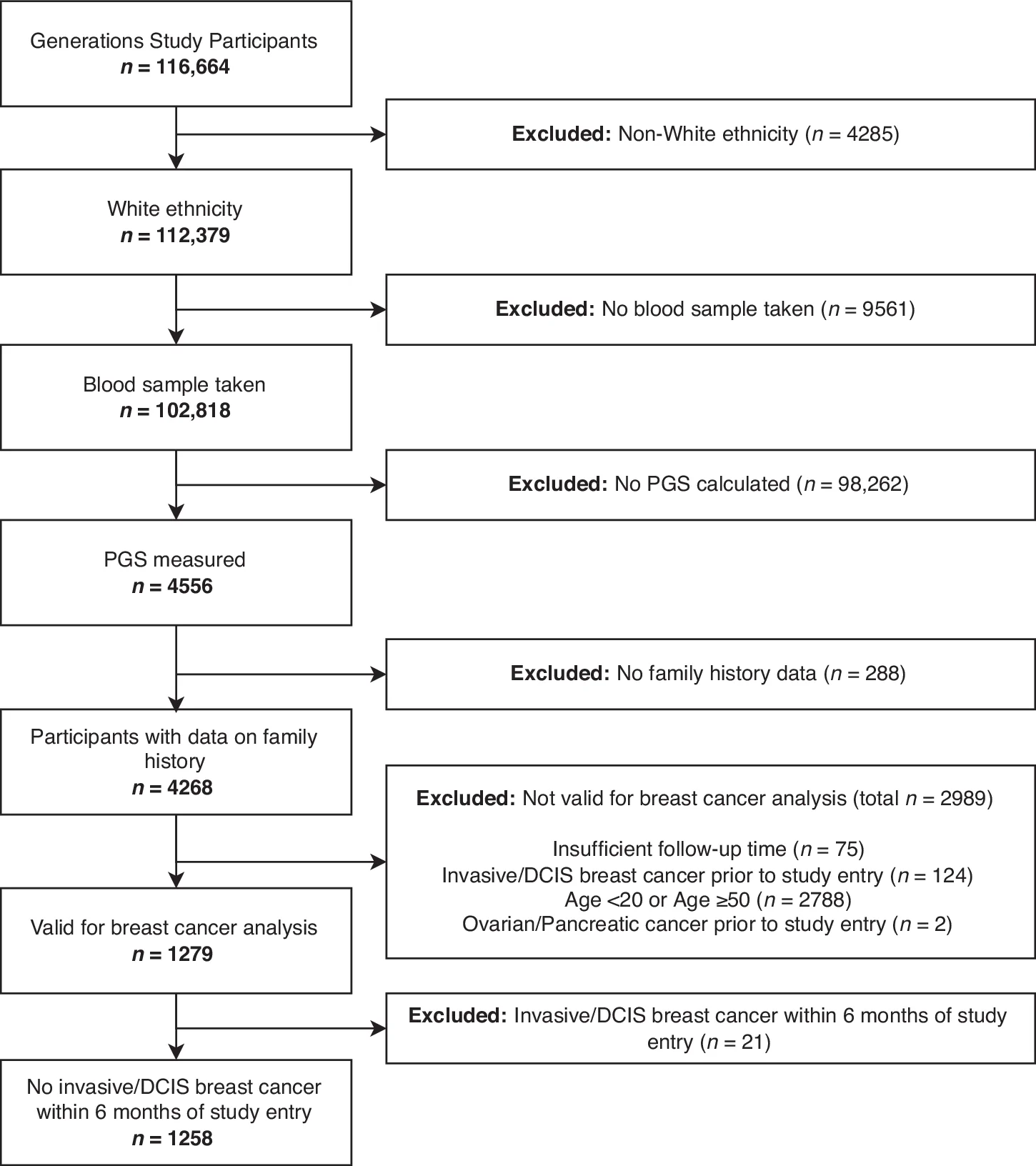
Flowchart detailing the study population selection process. DCIS ductal carcinoma in situ, PGS polygenic risk score.

To be eligible for this analysis, participants were required to have the following: PGS-313 score measured, age between 20 and 50 at entry to the study (defined as the date of receipt of the baseline questionnaire), no prior diagnosis of invasive/ductal carcinoma in situ (DCIS) BC, ovarian or pancreatic cancer within 6 months of entry to the study, and self-reported data on family history of BC. Participants also needed to have at least 10 years of follow-up from study entry until the earliest of either the date of death, the date of mailing of the last completed follow-up questionnaire, or the date at which the individuals ‘flagging’ coverage through the NHS Central Register ended. The study population selection process, including the number of eligible and excluded subjects, is shown in Fig. 1.

To account for sampling into the nested case-control study, the nested case-control sample of GS with PGS scores was weighted to represent the subset of GS eligible for genotyping (those with White ethnicity and a blood sample provided at study entry). These weights were then applied to the study population eligible for this analysis. Weights were calculated using inverse probability weighting (IPW), with each participant weighted by the inverse of their inclusion probability. We estimated the probability of a participant being included in the study population given age at entry to the GS a (5-year categories) and year of first BC diagnosis b (5-year categories + control category) as the following:

$${p}_{a,b}=\,\frac{{n}_{a,b}}{{N}_{a,b}}$$

where ${n}_{a,b}$ is the number of participants with PGS scores measured with age at entry to the GS a and year of first BC diagnosis b, and ${N}_{a,b}$ is the number of participants eligible for genotyping with age at entry to the study a and year of first BC diagnosis b. The weight of the participant given the variables specified was then calculated as ${w}_{a,b}=1/{p}_{a,b}$ by IPW. To ensure stability of these weights, in the event that ${n}_{a,b} < 10$, the category was merged with categories adjacent by age at entry to the study until the criteria are met. If no categories adjacent by age at entry to GS were present, the category was merged with categories adjacent by year at first BC.

To account for differences between the study population drawn from the GS and the UK population, the study population was then further weighted to the UK population with respect to age and family history of BC. For this, the initial weights were incorporated into an iterative proportional fitting procedure with target marginal counts within groups defined by age (by 5-year age bands) and family history of BC (binary yes/no). Target marginals were calculated using data from the Health Survey for England and published data on family history (Supplementary Tables 1a and 1b) [16].

### Missing data

By study design, measurements of PGS are complete in the study population. For all other covariates, data were assumed to be missing at random. BOADICEA allows for missing risk factor information by constraining the total age-specific incidence, summed over all possible risk factors and genotypes, to agree with the population incidence [7, 17].

### Risk assessment and criteria for referral

**Fig. 2:**
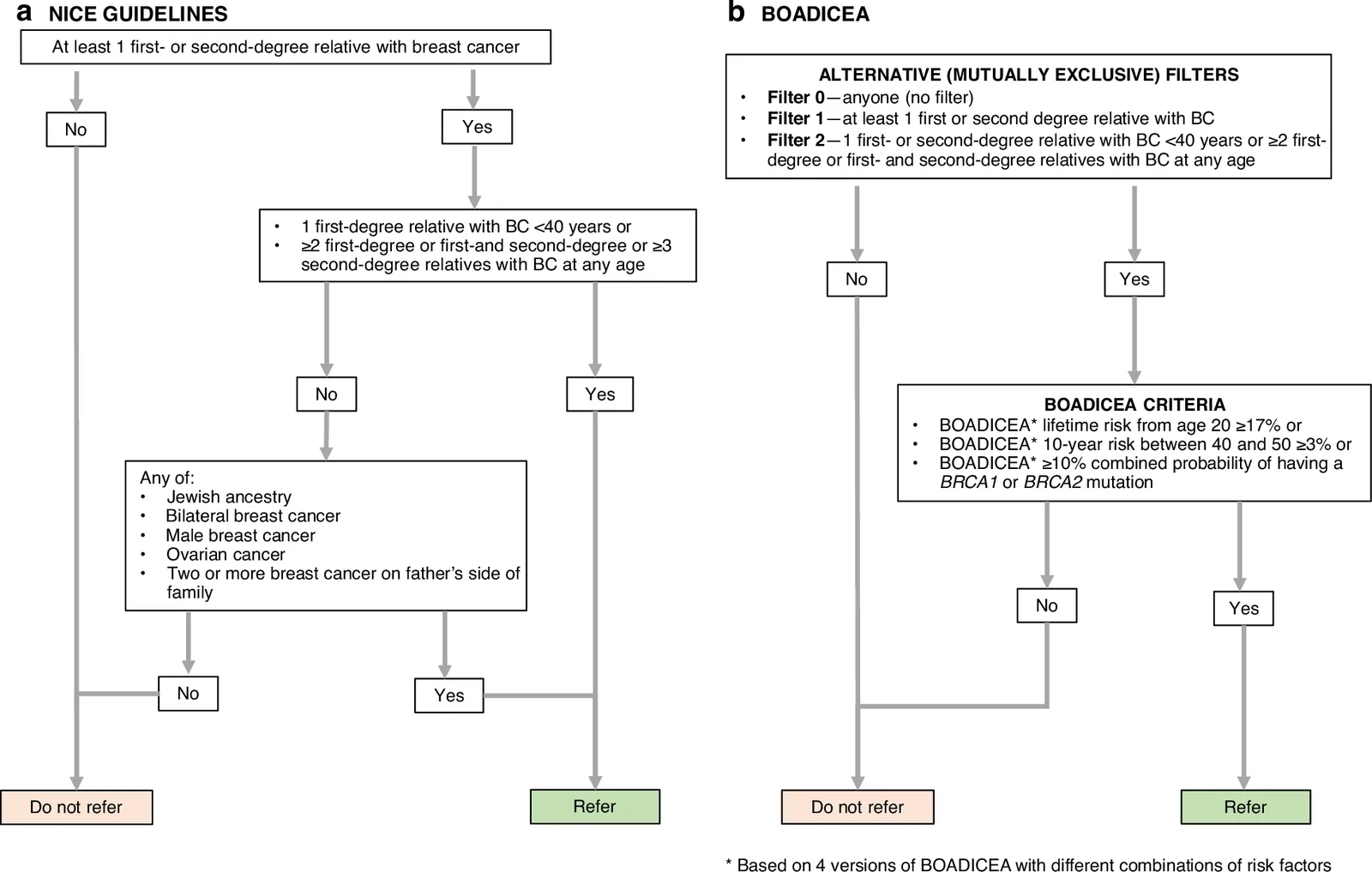
Criteria used for referral of women from primary care to secondary care. Guidelines flowchart for **a** NICE guidelines, **b** BOADICEA. BC breast cancer.

The criteria for referral of women from primary care to secondary care or clinical genetics services were based on either (i) the family history criteria within current NICE guidelines for use in primary care or (ii) the risk thresholds included within current NICE guidelines for use in secondary care and clinical genetics following risk assessment using the BOADICEA model [2] (Fig. 2). In both cases family history was based on self-report at baseline with no verification, mirroring what currently happens in primary care, and it was assumed that all women completed the risk assessment.

The NICE criteria (Fig. 2a) for people with a family history of BC are based on first and second-degree family history of BC, ovarian cancer, sarcomas, gliomas, and childhood adrenal cortical carcinomas, as well as Jewish ancestry. Data on family history of sarcomas, gliomas, and childhood adrenal cortical carcinomas were not collected for the study population, and so the family history criteria were based only on breast and ovarian cancer.

The criteria for referral based on the BOADICEA (v6) BC risk prediction model [17] (Fig. 2b) were those recommended in the NICE guidelines: ≥17% lifetime risk of BC, ≥3% risk of BC between the ages of 40 and 50, or ≥10% combined probability of having a *BRCA1* or *BRCA2* mutation. BOADICEA scores were generated for the study population, using data on risk factors, family history of cancer collected at study entry, and PGS. BOADICEA was originally developed using segregation analysis methods[7, 18] and therefore explicitly incorporates family history, including exact family relationships, ages at diagnosis for affected relatives, and ages at last observation for unaffected relatives. To evaluate the effect of including information on unaffected relatives, we generated BOADICEA risk scores by including and excluding unaffected family members. Similarly, to evaluate the impact of including PGS, we generated BOADICEA risk scores by including or excluding the PGS.

To evaluate a staged approach to risk assessment in which not all women in primary care are offered a full risk assessment, we additionally implemented two filters for the strategies incorporating the BOADICEA model (Fig. 2b). The first filter (Filter 1) offers a full risk assessment, and therefore the option of referral, only to women with a self-reported first or second-degree family history of BC. The second filter (Filter 2) offers full risk assessment and referral only to women with either a first- or second-degree relative diagnosed with BC at an age of less than 40 years old, or two or more first- or second-degree relatives with a history of BC. We denote the case where all women are offered full risk assessment as ‘Filter 0’. All filter options were implemented for all four risk factor combinations considered for calculating the BOADICEA risk scores. As current NICE guidelines for assessment in primary care recommend only obtaining a first and second-degree family history from women with a first or second-degree relative with BC, no additional filters were evaluated when using the NICE criteria, but only those women with a first or second-degree relative with BC are considered to undergo a full risk assessment.

### Statistical analysis

We first assessed the performance of the BOADICEA model within the fully weighted study population. Discrimination and calibration statistics for 10-year absolute risk from the four selected BOADICEA model uses were calculated using the fully weighted study population and the iCARE package in R [19]. Calibration was assessed by comparing the expected and observed number of cases (both overall and by decile of 10-year absolute risk) and the calibration slope. Discrimination was assessed using the area under the curve (AUC) for 10-year BC status.

To compare the different risk assessment strategies, we evaluated the ability of each strategy to identify individuals who were estimated to be at above population-level risk by the most complete BOADICEA model (including unaffected relatives and PGS, with no filter applied) and to identify individuals who develop BC within 10 years. For each strategy we report the percentage of the population who would undergo a risk assessment, the percentage of the population who would meet the referral criteria, the sensitivity (% of women who were estimated to be above population-level risk based on the most complete BOADICEA model or % of women who developed BC within 10 years who were referred under that strategy), the specificity (% of women who were estimated to be at population-level risk based on the most complete BOADICEA model or did not develop BC within 10 years who were not referred under that strategy), the precision (% of those referred by that strategy that were estimated to be above population-level risk based on the most complete BOADICEA model or who developed BC within 10 years) and the number of women needed to be referred and risk assessed to identify one woman either estimated to be above population-level risk based on the most complete BOADICEA model or who developed BC within 10 years. Confidence intervals for relevant metrics were estimated using bootstrapping (*N* = 10,000). All analyses were conducted in R.

### Sensitivity analyses

The comparison between non-PGS BOADICEA referral criteria and the NICE guidelines was also conducted in the unweighted source population from which the nested case-control sample was sampled. To assess the impact of missing data on age at menarche, we also repeated the analyses, including only women with known age at menarche.

### Ethics approval and consent to participate

All methods were performed in accordance with the relevant guidelines and regulations. The Generations Study was approved under the procedures for national medical research studies by the South-East Multi-Centre Research Ethics Committee (REC reference number 03/1/014). Informed consent was obtained from all participants. No additional approvals were required for this study.

## Results

### Description of the cohort

A total of 1258 women were eligible for inclusion in the analysis (Fig. 1). Among these, 392 developed BC (338 invasive BC and 54 DCIS) within the 10-year follow-up period. The characteristics of those women and the study population as a whole are given in Table 1 (weighted to the UK population) and Supplementary Table 2 (unweighted).

**Table 1:** Characteristics of the study population drawn from women aged between 20 and 50 at entry to the Generations Study between 2004 and 2011.

	Whole cohort	10-year BC cases
Characteristic	*N* = 1258	*N* = 392
Age (years), Mean (SD)	34.5 (8.3)	41.2 (6.2)
First-degree family history of breast cancer		
No	91%	80%
Yes	9%	20%
Second-degree family history of breast cancer		
No	98%	99%
Yes	2%	1%
Follow-up time (years), Mean (SD)	13.7 (1.4)	12.3 (2.4)
Age at menarche (years), Mean (SD)	12.6 (1.4)	12.7 (1.4)
Unknown	151	48
Parity, Mean (SD)	0.98 (1.07)	1.55 (1.02)
Unknown	14	9
Age at 1st birth (years), Mean (SD)	28.0 (4.8)	28.5 (5.2)
Unknown	559	93
OC usage		
Current	30%	17%
Not current	70%	83%
Unknown	14	9
HRT usage		
Current combined therapy	0%	1%
Current oestrogen therapy	1%	1%
Not Current	98%	97%
Unknown	19	12
BMI (kg/m²), Mean (SD)	24.4 (4.3)	25.3 (4.9)
Unknown	60	18
Alcohol intake (g/day), Mean (SD)	13.5 (14.3)	14.0 (16.3)
Unknown	15	9
Age at menopause (years), Mean (SD)	41.9 (5.9)	43.2 (5.2)
Unknown	1201	362
Height (cm), Mean (SD)	166 (7)	165 (7)
Unknown	18	10
PGS (raw), Mean (SD)	−0.36 (0.60)	−0.14 (0.59)
PGS (standardised), Mean (SD)	0.11 (0.99)	0.46 (0.97)

To be eligible, participants were required to have no prior diagnosis of invasive breast cancer, ductal carcinoma in situ, ovarian or pancreatic cancer within 6 months of entry to the study, data on polygenic risk of breast cancer, self-reported data on family history of breast cancer, and 10 years of follow-up. The study population was then weighted to represent the UK population with respect to age and family history of breast cancer.

*BC* breast cancer, *SD* standard deviation, *PGS* polygenic score.

### Calibration and discrimination of the BOADICEA model

Table 2 shows the results of the calibration and discrimination analysis in the weighted study population for the four BOADICEA model uses we tested. All four risk factor combinations were found to have good discrimination, with AUCs ranging from 0.74 (95% CI: 0.70–0.78) for the model without unaffected family members and without PGS to 0.76 (95% CI: 0.72–0.79) for the model including both unaffected family members and PGS (Supplementary Fig. 1). Slight underestimation of risk was found with total expected/observed ratios ranging between 0.78-0.85 for all four combinations. Inclusion of PGS improved the calibration slope across all risk factor combinations, both with and without unaffected family members (Supplementary Fig. 2).

**Table 2:** Discrimination and calibration for four complexities of the BOADICEA model, including different combinations of information for predicting 10-year absolute risk.

Including unaffected family members	Including PGS	AUC (95% CI)	E/O (95% CI)	Calibration slope (95% CI)
FALSE	FALSE	0.74 (0.70, 0.78)	0.83 (0.70, 0.98)	1.29 (0.90, 1.67)
TRUE	FALSE	0.74 (0.70, 0.78)	0.78 (0.66, 0.92)	1.34 (0.87, 1.80)
FALSE	TRUE	0.76 (0.72, 0.79)	0.85 (0.72, 1.01)	1.09 (0.86, 1.31)
TRUE	TRUE	0.76 (0.72, 0.79)	0.81 (0.68, 0.96)	1.14 (0.90, 1.37)

*PGS* polygenic score, *AUC* area under the curve, *E/O* expected/observed ratio, *CI* confidence interval.

### Comparison with the full BOADICEA model as the gold standard

Table 3 shows the proportion of women who would be referred using each risk assessment strategy and the sensitivity and specificity of each strategy when compared with the full BOADICEA model as the gold standard. Applying the gold standard full BOADICEA model, incorporating affected and unaffected relatives and PGS, to the whole population in primary care would result in 26.5% of women under age 50 meeting the referral criteria and being categorised as at above-population level risk, the majority (25.2%) based on lifetime risk (Supplementary Fig. 3). In this scenario, 3.8 women needed to be assessed in primary care to identify one woman at above-population level risk.

**Table 3:** Comparison of referral processes by NICE guidelines and different versions of the BOADICEA model to identify women estimated to be at above population level risk based on the most complete BOADICEA model, including unaffected family members (UFM) and polygenic scores (PGS) with no filter prior to risk assessment (filte﻿r 0).

Model	Including unaffected family members	Including PGS	Filter question^a^	Full risk assessment (%) (95% CI)	Referred (%) (95% CI)	Sensitivity (%) (95% CI)^b^	Specificity (%) (95% CI)^c^	Precision (%) (95% CI)^d^	Number of risk assessments for one woman estimated to be at above population-level risk to be referred (95% CI)
NICE	FALSE	FALSE	-	11.0 (9.1, 13.1)	1.4 (0.7, 2.2)	4.5 (2.2, 7.3)	99.7 (99.2, 100.0)	83.4 (59.8, 99.9)	9.3 (5.9, 18.6)
BOADICEA	TRUE	TRUE	0	100.0 (100.0, 100.0)	26.5 (23.1, 30.1)	Reference	Reference	Reference	3.8 (3.3, 4.3)
BOADICEA	TRUE	TRUE	1	11.0 (9.1, 13.1)	6.7 (5.2, 8.4)	25.3 (19.9, 31.4)	100.0 (100.0, 100.0)	100.0 (100.0, 100.0)	1.6 (1.4, 1.9)
BOADICEA	TRUE	TRUE	2	1.5 (0.7, 2.3)	1.2 (0.6, 2.0)	4.6 (2.1, 7.5)	100.0 (100.0, 100.0)	100.0 (100.0, 100.0)	1.2 (1.0, 1.7)
BOADICEA	TRUE	FALSE	0	100.0 (100.0, 100.0)	11.9 (9.8, 14.3)	29.5 (23.4, 36.3)	94.4 (92.5, 96.2)	65.7 (56.2, 75.0)	12.8 (10.2, 16.4)
BOADICEA	TRUE	FALSE	1	11.0 (9.1, 13.1)	8.3 (6.7, 10.1)	22.6 (17.5, 28.4)	96.8 (95.5, 98.0)	71.9 (62.0, 81.0)	1.8 (1.6, 2.2)
BOADICEA	TRUE	FALSE	2	1.5 (0.7, 2.3)	1.5 (0.7, 2.3)	4.6 (2.1, 7.5)	99.6 (99.1, 100.0)	81.8 (57.7, 99.7)	1.2 (1.0, 1.7)
BOADICEA	FALSE	TRUE	0	100.0 (100.0, 100.0)	28.3 (24.9, 31.9)	100.0 (100.0, 100.0)	97.5 (96.1, 98.7)	93.6 (90.0, 96.5)	3.8 (3.3, 4.3)
BOADICEA	FALSE	TRUE	1	11.0 (9.1, 13.1)	6.8 (5.3, 8.5)	25.3 (19.9, 31.4)	99.8 (99.5, 100.0)	98.1 (94.8, 100.0)	1.6 (1.4, 1.9)
BOADICEA	FALSE	TRUE	2	1.5 (0.7, 2.3)	1.2 (0.6, 2.0)	4.6 (2.1, 7.5)	100.0 (100.0, 100.0)	99.9 (99.5, 100.0)	1.2 (1.0, 1.7)
BOADICEA	FALSE	FALSE	0	100.0 (100.0, 100.0)	13.9 (11.6, 16.4)	31.5 (25.2, 38.4)	92.5 (90.2, 94.5)	60.1 (50.8, 69.1)	12.0 (9.6, 15.3)
BOADICEA	FALSE	FALSE	1	11.0 (9.1, 13.1)	9.0 (7.3, 10.9)	22.7 (17.6, 28.5)	96.0 (94.5, 97.3)	67.1 (57.2, 76.6)	1.8 (1.6, 2.2)
BOADICEA	FALSE	FALSE	2	1.5 (0.7, 2.3)	1.5 (0.7, 2.3)	4.6 (2.1, 7.5)	99.6 (99.1, 100.0)	81.8 (57.7, 99.7)	1.2 (1.0, 1.7)

^a^Filter 0: all participants remain in the referral process. Filter 1: participants without a family history of breast cancer removed from the referral process. Filter 2: participants without multiple family members with a history of breast cancer or one family member with breast cancer diagnosed before the age of 40, removed from the referral process.

^b^% of women estimated to be above population-level risk based on the most complete BOADICEA model (reference) who were referred under that process.

^c^% of women estimated to be at population-level risk based on the most complete BOADICEA model (reference), who were not referred under that process.

^d^% of women who were referred under that process estimated to be above population-level risk based on the most complete BOADICEA model (reference).

By comparison, applying the current NICE criteria to the whole population in primary care would result in 1.4% of the population meeting criteria for referral. Of those women referred, 83% would be categorised as above-population-level risk based on the full BOADICEA model (Supplementary Fig. 4). However, they would represent only 4.5% of the population classified as being above population-level risk based on the full BOADICEA model, and nearly three times as many women (*n* = 9.3) would need to be assessed to identify one woman at above population-level risk.

Restricting risk assessment with the full BOADICEA model to only women with a family history of BC in first- or second-degree relatives (Filter 1—11% of the population) would result in less women being referred than when the full model is offered to all women (6.7 vs 26.5%) and less women needing to be risk assessed to find one woman at above population level risk (*n* = 1.6 vs *n* = 3.8); it would identify 25.3% of those at above-population level risk based on the full BOADICEA model.

Restricting risk assessment with the full BOADICEA model to only those with a significant family history of BC (Filter 2—1.5% of the population) further reduces the proportion of women being referred to 1.2% and the number of women needed to be risk assessed to find one woman at above population level risk to 1.2; however, it would identify only 4.6% of those at above-population level risk based on the full BOADICEA model. This sensitivity of 4.6% is comparable to that seen with the current NICE criteria (4.5%) but is achieved with many fewer women needing to be risk assessed to identify one woman at above-population-level risk than with the current NICE criteria (*n* = 1.2 vs *n* = 9.3).

Removing PGS from the full BOADICEA model decreases the proportion of women referred from 26.5 to 11.9% when the whole population is risk assessed but results in the highest number of women needed to be risk assessed to find one women at above population level risk across all considered strategies (*n* = 12.8) and would only identify 29.5% of those women at above-population level risk based on the full BOADICEA model including PGS. When the risk assessment is restricted to those with a family history (Filter 1), the differences between the full BOADICEA model and the BOADICEA model without PGS are smaller, and when the risk assessment is restricted to only those with a significant family history (Filter 2), removal of the PGS has no measurable effect on sensitivity.

Removal of unaffected relatives from the BOADICEA model does not affect sensitivity. With no family history filter or Filter 1, removal of unaffected relatives results in a small increase in the proportion of women referred, a small decrease in specificity and a small increase in the number needed to refer to identify a woman at above-population level risk. Notably, in this strategy, 96% of women referred did not meet the NICE criteria for referral (Supplementary Fig. 4). When risk assessment is restricted to those with a strong family history (Filter 2), the removal of unaffected relatives has no measurable impact on any metric.

### Comparison with 10-year breast cancer status

Table 4 shows the ability of each considered strategy to identify those women who develop BC within 10 years. The 26.5% of women referred based on risk assessment of the whole population with the full BOADICEA model would include 34.8% of the women who develop BC within 10 years, with 185 women needing to be assessed to identify one woman who would develop BC.

**Table 4:** Comparison of referral processes in identifying breast cancer cases occurring within 10 years since risk assessment or referral.

Model	Including unaffected family members	Including PGS	Filter question^a^	Full risk assessment (%) (95% CI)	Referred (%) (95% CI)	Sensitivity (%) (95% CI)^b^	Specificity (%) (95% CI)^c^	Precision (%) (95% CI)^d^	Number of risk assessments for one 10-year BC case to be referred (95% CI)
NICE	FALSE	FALSE	-	11.0 (9.1, 13.1)	1.4 (0.7, 2.2)	4.4 (2.4, 7.1)	98.6 (97.8, 99.3)	4.8 (2.2, 10.6)	160.1 (96.2, 310.1)
BOADICEA	TRUE	TRUE	0	100.0 (100.0, 100.0)	26.5 (23.1, 29.9)	34.8 (27.9, 41.9)	73.7 (70.1, 77.1)	2.0 (1.5, 2.7)	184.5 (145.2, 241.9)
BOADICEA	TRUE	TRUE	1	11.0 (9.1, 13.1)	6.7 (5.2, 8.4)	15.7 (10.9, 21.2)	93.4 (91.8, 95.0)	3.7 (2.3, 5.5)	44.8 (30.6, 68.4)
BOADICEA	TRUE	TRUE	2	1.5 (0.7, 2.4)	1.2 (0.5, 2.0)	3.7 (1.8, 6.3)	98.8 (98.0, 99.5)	4.8 (2.0, 11.7)	25.4 (11.1, 60.7)
BOADICEA	TRUE	FALSE	0	100.0 (100.0, 100.0)	11.9 (9.7, 14.2)	18.4 (12.9, 24.5)	88.2 (85.8, 90.4)	2.4 (1.6, 3.5)	349.5 (252.2, 517.2)
BOADICEA	TRUE	FALSE	1	11.0 (9.1, 13.1)	8.3 (6.6, 10.1)	15.7 (10.9, 21.3)	91.8 (89.9, 93.5)	3.0 (1.9, 4.4)	44.8 (30.6, 68.6)
BOADICEA	TRUE	FALSE	2	1.5 (0.7, 2.4)	1.5 (0.7, 2.4)	4.0 (2.0, 6.6)	98.6 (97.7, 99.3)	4.2 (1.9, 9.5)	23.8 (10.6, 53.8)
BOADICEA	FALSE	TRUE	0	100.0 (100.0, 100.0)	28.3 (24.8, 31.9)	37.1 (30.0, 44.3)	71.8 (68.2, 75.4)	2.0 (1.5, 2.6)	173.1 (137.4, 224.8)
BOADICEA	FALSE	TRUE	1	11.0 (9.1, 13.1)	6.8 (5.3, 8.5)	15.9 (11.1, 21.4)	93.3 (91.6, 94.9)	3.6 (2.3, 5.4)	44.4 (30.4, 67.4)
BOADICEA	FALSE	TRUE	2	1.5 (0.7, 2.4)	1.2 (0.6, 2.0)	3.8 (1.9, 6.4)	98.8 (98.0, 99.5)	4.9 (2.1, 11.9)	24.8 (10.9, 58.3)
BOADICEA	FALSE	FALSE	0	100.0 (100.0, 100.0)	13.9 (11.5, 16.4)	19.2 (13.7, 25.3)	86.2 (83.7, 88.6)	2.2 (1.4, 3.1)	333.9 (244.5, 486.0)
BOADICEA	FALSE	FALSE	1	11.0 (9.1, 13.1)	9.0 (7.2, 10.9)	16.3 (11.4, 21.9)	91.1 (89.2, 93.0)	2.8 (1.9, 4.2)	43.3 (29.8, 65.5)
BOADICEA	FALSE	FALSE	2	1.5 (0.7, 2.4)	1.5 (0.7, 2.4)	4.0 (2.0, 6.6)	98.6 (97.7, 99.3)	4.2 (1.9, 9.5)	23.8 (10.6, 53.8)

^a^Filter 0: all participants remain in the referral process. Filter 1: participants without a family history of breast cancer removed from the referral process. Filter 2: participants without multiple family members with a history of breast cancer or one family member with breast cancer diagnosed before the age of 40 removed from the referral process.

^b^% of women who developed breast cancer within 10 years who were referred under that process.

^c^% of women who did not develop breast cancer within 10 years who were not referred under that process.

^d^% of women who were referred under that process who developed breast cancer within 10 years.

Referral processes differ by inclusion/exclusion of unaffected family members (UFM) and polygenic scores (PGS), and filtering strategies.

By comparison, the 1.4% of the population referred based on the current NICE criteria would include only 4.4% of those women who develop BC and 161 women would need to be assessed to identify one woman who would develop BC.

The characteristics of the women who developed BC and were or were not identified by either approach are given in Supplementary Table 3. Compared with all the women who developed BC, women not referred by either approach were less likely to have a first- or second-degree family history of BC (7 vs 21%) and had a lower mean standardised PGS (0.12 (SD 0.83) vs 0.46 (SD 0.97)).

Restricting the full BOADICEA risk assessment to only those with a family history of BC (Filter 1) identifies approximately half of those who develop BC, which are identified when risk assessment is performed for the whole population (15.7 vs 34.8%). However, it would require a much smaller proportion of women needing to be risk assessed (11 vs 100%) or referred (6.7 vs 26.5%), and the number of women needing to be assessed to refer one woman who would subsequently develop BC would decrease from 185 to 45. Restricting even further to only those with a strong family history of BC (Filter 2) reduces the proportion of women who subsequently develop BC who are identified as above population-level risk to lower than using the NICE criteria (3.7 vs 4.5%) but also decreases the number of women needing to be risk assessed to identify one woman who develops BC to well below the number obtained when using the NICE criteria (*n* = 26 vs *n* = 161).

As seen in the analysis with the full BOADICEA model as the gold standard, removal of PGS from the full BOADICEA model when risk assessing the whole population decreases the proportion of women referred but results in the highest number of women needing to be risk assessed to find one women who develops BC across all considered strategies (*n* = 350) and identifies fewer women who subsequently develop BC (18.4 vs 34.8% for the BOADICEA model including PGS or without PGS respectively). Conversely, removing PGS from the BOADICEA model when risk assessment is only offered to women with a family history of BC (Filter 1) or a strong family history (Filter 2) results in slightly more women being referred, a slightly increased sensitivity, and a similar number needing to be assessed to identify one woman who would subsequently develop BC.

Removal of unaffected relatives makes little difference to any of the considered metrics, whether the whole population is risk assessed, or risk assessment is limited to those with a family history. In all cases, removal of unaffected relatives results in a small increase in the proportion referred, a small increase in the proportion of women who subsequently develop BC identified and a small decrease in the number needing to be risk assessed to identify one case.

### Sensitivity analyses

Both sensitivity analyses yielded similar results to the main analysis (Supplementary Tables 4, 5a and 5b).

## Discussion

### Key findings

To our knowledge, this is the first study to directly compare the current primary care NICE family history-based risk assessment and referral criteria with multifactorial BC risk assessment in a population-based cohort. We show that applying the current NICE approach to the whole population of UK women under age 50 misses over 95% of women estimated to be at above-population level risk based on multifactorial risk assessment using the BOADICEA model, and over 95% of women who go on to develop BC within 10 years. We estimate that if the full BOADICEA model were used, seven times as many women would be identified as eligible for early detection and preventative interventions—potentially detecting earlier or preventing up to an additional 3000 BC cases over current NICE guidelines [20]. We further show that 73% of women under 50 years of age at the time of risk assessment and estimated to be at above-population level risk based on the full BOADICEA model do not have a family history of BC and restricting risk assessment to only those women with a family history of BC reduces the number of women who subsequently develop BC identified by the full BOADICEA model by half from 34.8 to 15.7%.

The removal of unaffected relatives from the BOADICEA multifactorial risk assessment in this primary care population has little effect at a population level. Conversely, the removal of PGS from the BOADICEA model when all women are risk assessed results in a substantially reduced sensitivity to identify women who will develop BC over the following 10 years and a substantial increase in the number of women needing to be risk assessed to identify one woman at high risk or one woman who will develop BC. When the risk assessment is restricted to women with a family history of BC, the impact of removing the PGS at a population level is much smaller.

Our findings also highlight the trade-offs between identifying a greater proportion of women who are at estimated above-population risk or develop BC within 10 years and the resources needed within the healthcare system. While applying the current NICE guidance to all women under 50 would only identify <5% of those who develop BC, it requires only 1.4% of that population to be referred. In contrast, offering the full BOADICEA risk assessment to all women under 50 would identify 34.8% of those who develop BC, but would result in 26.5% of women potentially being referred to secondary care or clinical genetics services. This approach would have substantial resource implications for both primary care workflows and secondary care and genetics service capacity.

### Comparison with existing literature

Our findings are consistent with a previous study in women attending screening in the UK which demonstrated the added value of multifactorial BC risk assessment over NICE family history-based criteria [21]; the NICE criteria classified 3.7% of women aged 46–49 years as at above-population level risk while the Tyrer-Cuzick model incorporating family history, hormonal and reproductive, and lifestyle factors classified 8.8% as at above-population level risk [21]. The performance of the BOADICEA model to identify younger women who later develop BC and the importance of PGS in this group has also been demonstrated within the UK Biobank cohort [22]. Using the NICE 10-year risk threshold with the BOADICEA model incorporating first-degree family history, questionnaire-based risk factors (QRFs), and PGS identified 25.4% of women under 50 at above-population level risk, with 44.2% of BC patients being in that group. Removing PGS reduced those proportions to 11.3 and 17.4%, respectively [22]. These figures are similar to our findings, with the differences likely explained by the inclusion of only first-degree relatives in that earlier study and the higher incidence of BC amongst UK Biobank participants.

### Strengths and limitations

The added value of our study over these previous studies is that we have directly compared the current NICE family history-based criteria to a series of strategies incorporating one of the most widely validated multifactorial risk models, the BOADICEA model, amongst a population-based cohort of women aged under 50 at the time of recruitment. By using the GS cohort, we were able to include both first- and second-degree family history of BC and weighting the study population to the UK population with respect to age and family history of BC allowed us, at least partially, to account for any recruitment bias. Our approach is not without limitations, though. Specifically, we were not able to include family history of ovarian cancer beyond parents, grandparents, siblings and children, and no data were available on family history of sarcomas, gliomas, and childhood adrenal cortical carcinomas, which are included in current NICE guidelines as additional reasons for referral amongst women with a first- or second-degree relative with BC. Mammographic breast density and rare pathogenic variants were also not available in the dataset, and we only compared the NICE guidelines to one multifactorial BC risk model. While the BOADICEA model is one of the most widely used in the UK and is recommended in NICE guidelines, other models exist that could be used for this purpose [23]. The number of women included in some of the comparisons, most notably those where risk assessment is limited to the 1.5% of women with a strong family history, is also small. The impact of removing the PGS and unaffected relatives in these women, in particular, must therefore be interpreted with caution. We also only include White women. Additional studies will be needed for other races and ethnicities, incorporating the recent ethnicity-adapted version of BOADICEA [24].

Additionally, we have assumed that all those eligible for a risk assessment in each of the strategies undergo that risk assessment. While BC risk assessment has been shown to be widely acceptable amongst the public [25–32], the only published study offering BC risk assessment within primary care in the UK had an uptake of 16.1% [33]. Decreased uptake would not affect the numbers needed to be assessed to identify one woman at above-population level risk or one BC case. The sensitivity of each strategy would be reduced but proportionately across all strategies, meaning that the absolute benefits of the BOADICEA-based strategies would be reduced, but the relative benefits would be the same. Our analysis also focuses on the identification of higher-risk women. The resultant downstream clinical benefits of identifying these women will depend on the uptake and effectiveness of early detection and preventive interventions.

### Implications

Previous studies have shown that sensitivity is valued more than specificity amongst the public despite the potential for patient anxiety and unnecessary referrals [34, 35]. Based on the results for 10-year BC status, the most preferred strategy on that basis alone would be the full BOADICEA risk assessment without considering unaffected relatives, offered to all women under 50. This strategy would identify 37% of women who would develop BC in the following 10 years. However, this strategy also requires the most risk assessments, both in primary care and in secondary care or genetics services, has the lowest specificity and hence the highest number of women needing to be referred to identify a case, and 174 women needing to be risk assessed to find one woman who would subsequently develop BC within 10 years—more than the current NICE criteria.

If, instead, the priority is to reduce the number needed to be risk assessed in primary care to identify a woman who would develop BC in the next 10 years, then restricting the BOADICEA risk assessment to those with a significant family history would be the preferred option. In that group, our findings suggest that PGS may add little to the outcome at a population level, so the risk assessment could be based on just QRFs and family history. Such a strategy would require only 25 women to be risk assessed to find a case. However, it would also only identify 3.8% of women who subsequently develop BC, and so the potential for false reassurance amongst women would need to be carefully considered. Notably, though, it would be more efficient than the current NICE criteria, which would identify a similar proportion of women who subsequently develop BC (4.4%) but would require nearly seven times as many women to be assessed to identify one woman who would develop BC.

A middle ground would be to offer the BOADICEA risk assessment without considering unaffected relatives only to those women with a first- or second- degree family history of BC. This is also the most direct comparison to current care, in which risk assessments are offered in primary care to women who self-present with concerns about their family history. Compared with offering the full BOADICEA risk assessment to all women, this would reduce the number of risk assessments to 11% of the population, reduce the proportion of women referred from 27 to 7%, halve the number of women referred to find a case from 50 to 27 and reduce the number needed to refer to identify a woman who would subsequently develop BC from 185 to 45. This would, however, be at the expense of identifying only 25% of those estimated to be at above-population risk and halving the sensitivity of finding women who develop BC from 35 to 16%. Compared with offering the same women risk assessment with the NICE guidelines, this would also result in more referrals (6.7 vs 1.4%) but would identify almost four times as many women who would go on to develop BC (15.7 vs 4.4%), with only 45 women needing to be risk assessed to identify one woman who subsequently develops BC, compared with 161 for the NICE criteria.

## Conclusions

This study demonstrates that the current NICE family history-based criteria applied to all women under 50 miss 95% of women estimated to be at above-population level risk based on multifactorial risk assessment and 95% of those who go on to develop BC within 10 years. Multifactorial risk assessment could identify up to 35% of women who develop BC while also reducing the number needed to be assessed to identify one woman who later develops BC. However, many of the multifactorial risk assessment strategies considered would require substantial resources for both risk assessment and subsequent assessment within secondary care and clinical genetics services. Ultimately, the decision around whom to offer risk assessment to and which risk factors to include will be a trade-off between the practical, resource, and cost implications of data collection and risk assessment, and the potential benefits and harms associated with accurate and inaccurate classification of women. On-going studies evaluating the feasibility and cost-effectiveness of multifactorial BC risk assessment within primary care [36–38] will help to inform these decisions, and further work is needed to evaluate the downstream impact on genetics and secondary care services and the longer-term impact of identifying these women on clinical outcomes. Alternative risk models or risk thresholds for multifactorial risk assessment might also achieve an even more favourable balance between sensitivity and specificity.

## Supplementary information

Supplementary Information

## Data Availability

All data relevant to this report are included in this published article and its supplementary information files. The access policy for analysis of the Generations study datasets is available at: https://www.breakthroughgenerations.org.uk/researchers.

## Code availability

The statistical code used for the analysis is available from the authors on request.
