# Supplementary material for: Comparison of NICE criteria with the BOADICEA multifactorial risk model to guide breast cancer risk assessment and referral amongst women under age 50 within primary care
Source: Br J Cancer. 2026 Aug 4;135(9):1452–62. doi: 10.1038/s41416-026-03547-2 (PMC13675180; doi:10.1038/s41416-026-03547-2)
Supplement: Supplementary file 1 [file 41416_2026_3547_MOESM1_ESM.docx]

**Supplementary Information**

**Supplementary Table 1a:** Target distribution of family history of breast cancer used in weighting the study sample.

| Category | Percentage (%) |
| --- | --- |
| No family history of breast cancer | 89.00 |
| Family history of breast cancer | 11.00 |

**Supplementary Table 1b:** Target distribution age used in weighting the study sample.

| Category | Percentage (%) |
| --- | --- |
| [20,25) | 15.14 |
| [25,30) | 16.62 |
| [30,35) | 18.19 |
| [35,40) | 17.88 |
| [40,45) | 16.94 |
| [45,50) | 15.23 |

**Supplementary Table 2: Characteristics of the unweighted study population drawn from women aged between 20 and 50 at entry to the Generations Study between 2004 and 2011. BC – breast cancer; SD – standard deviation; PGS – polygenic score.**

|  | **Whole Cohort** | **10-year BC Cases** |
| --- | --- | --- |
| **Characteristic** | **N = 1,258** | **N = 392** |
| Age (years), Mean (SD) | 36.1 (7.8) | 42.3 (5.5) |
| First degree family history of breast cancer |  |  |
| No | 83% | 74% |
| Yes | 17% | 26% |
| Second degree family history of breast cancer |  |  |
| No | 97% | 98% |
| Yes | 3% | 2% |
| Follow-up time (years), Mean (SD) | 13.4 (1.9) | 12.3 (2.5) |
| Age at menarche (years), Mean (SD) | 12.6 (1.4) | 12.7 (1.5) |
| Unknown | 151 | 48 |
| Parity | 1.08 (1.09) | 1.61 (1.05) |
| Unknown | 14 | 9 |
| Age at 1st birth (years), Mean (SD) | 27.8 (4.6) | 27.9 (4.8) |
| Unknown | 559 | 93 |
| OC usage |  |  |
| Current | 28% | 16% |
| Not Current | 72% | 84% |
| Unknown | 14 | 9 |
| HRT usage |  |  |
| Current combined therapy | 0% | 1% |
| Current oestrogen therapy | 1% | 2% |
| Not Current | 98% | 97% |
| Unknown | 19 | 12 |
| BMI (kg/m²), Mean (SD) | 24.6 (4.5) | 25.2 (4.7) |
| Unknown | 60 | 18 |
| Alcohol intake (g/day), Mean (SD) | 13.5 (14.9) | 14.4 (15.7) |
| Unknown | 15 | 9 |
| Age at menopause (years), Mean (SD) | 42.7 (5.4) | 42.6 (5.4) |
| Unknown | 1,201 | 362 |
| Height (cm), Mean (SD) | 165 (7) | 165 (7) |
| Unknown | 18 | 10 |
| PRS (raw), Mean (SD) | -0.31 (0.61) | -0.18 (0.61) |
| PRS (standardised), Mean (SD) | 0.19 (1.00) | 0.40 (0.99) |

**Supplementary Table 3: Characteristics of all women who developing breast cancer within 10-years and those who would have been referred/not referred based on the NICE criteria, the BOADICEA multifactorial model (Filter 0) + PRS + unaffected family members, or either. Study population drawn from women aged between 20 and 50 at entry to the Generations Study between 2004 and 2011. BC – breast cancer; SD – standard deviation; PGS – polygenic score.**

|  | **All 10-year BC Cases** | ***NICE*** | | ***BOADICEA^1^*** | | ***NICE or BOADICEA^1^*** | |
| --- | --- | --- | --- | --- | --- | --- | --- |
|  |  | **Referred** | **Not Referred** | **Referred** | **Not Referred** | **Referred** | **Not Referred** |
| **Characteristic** | **N = 392** | **N = 26** | **N = 366** | **N = 143** | **N = 249** | **N = 146** | **N = 246** |
| Age (years), Mean (SD) | 41.2 (6.2) | 37.7 (7.0) | 41.3 (6.1) | 38.7 (7.1) | 42.5 (5.2) | 38.7 (7.1) | 42.5 (5.2) |
| First degree family history of breast cancer |  |  |  |  |  |  |  |
| No | 80% | 0% | 84% | 56% | 93% | 56% | 93% |
| Yes | 20% | 100% | 16% | 44% | 7% | 44% | 7% |
| Second degree family history of breast cancer |  |  |  |  |  |  |  |
| No | 99% | 100% | 99% | 99% | 100% | 99% | 100% |
| Yes | 1% | 0% | 1% | 1% | 0% | 1% | 0% |
| Follow-up time (years), Mean (SD) | 12.3 (2.4) | 11.2 (2.6) | 12.3 (2.4) | 12.3 (2.3) | 12.2 (2.5) | 12.3 (2.3) | 12.2 (2.5) |
| Age at menarche (years), Mean (SD) | 12.7 (1.4) | 12.3 (1.2) | 12.7 (1.4) | 12.4 (1.4) | 12.9 (1.5) | 12.4 (1.4) | 12.9 (1.5) |
| Unknown | 48 | 3 | 45 | 10 | 38 | 10 | 38 |
| Parity, Mean (SD) | 1.55 (1.02) | 1.38 (1.34) | 1.56 (1.01) | 1.20 (1.02) | 1.74 (0.97) | 1.21 (1.03) | 1.74 (0.97) |
| Unknown | 9 | 0 | 9 | 1 | 8 | 1 | 8 |
| Age at 1st birth (years), Mean (SD) | 28.5 (5.2) | 28.0 (4.5) | 28.5 (5.3) | 28.4 (4.5) | 28.6 (5.5) | 28.4 (4.5) | 28.6 (5.5) |
| Unknown | 93 | 7 | 86 | 41 | 52 | 41 | 52 |
| OC usage |  |  |  |  |  |  |  |
| Current | 17% | 11% | 17% | 20% | 15% | 20% | 15% |
| Not Current | 83% | 89% | 83% | 80% | 85% | 80% | 85% |
| Unknown | 9 | 0 | 9 | 1 | 8 | 1 | 8 |
| HRT usage |  |  |  |  |  |  |  |
| Current combined therapy | 1% | 0% | 1% | 1% | 1% | 1% | 1% |
| Current oestrogen therapy | 1% | 2% | 1% | 1% | 2% | 1% | 2% |
| Not Current | 97% | 98% | 97% | 98% | 97% | 98% | 97% |
| Unknown | 12 | 0 | 12 | 3 | 9 | 3 | 9 |
| BMI (kg/m²), Mean (SD) | 25.3 (4.9) | 23.7 (4.1) | 25.4 (4.9) | 25.6 (5.2) | 25.1 (4.7) | 25.6 (5.2) | 25.1 (4.7) |
| Unknown | 18 | 0 | 18 | 4 | 14 | 4 | 14 |
| Alcohol intake (g/day), Mean (SD) | 14.0 (16.3) | 19.9 (15.5) | 13.7 (16.3) | 19.8 (19.8) | 10.8 (13.0) | 19.8 (19.7) | 10.8 (13.0) |
| Unknown | 9 | 0 | 9 | 1 | 8 | 1 | 8 |
| Age at menopause (years), Mean (SD) | 43.2 (5.2) | 42.0 (-) | 43.2 (5.2) | 44.3 (3.3) | 43.0 (5.4) | 44.3 (3.3) | 43.0 (5.4) |
| Unknown | 362 | 25 | 337 | 138 | 224 | 141 | 221 |
| Height (cm), Mean (SD) | 165 (7) | 167 (7) | 165 (7) | 166 (6) | 164 (7) | 166 (6) | 164 (7) |
| Unknown | 10 | 0 | 10 | 1 | 9 | 1 | 9 |
| PRS (raw), Mean (SD) | -0.14 (0.59) | -0.28 (0.60) | -0.14 (0.59) | 0.25 (0.53) | -0.36 (0.51) | 0.25 (0.54) | -0.35 (0.51) |
| PRS (standardised), Mean (SD) | 0.46 (0.97) | 0.24 (0.98) | 0.47 (0.97) | 1.11 (0.87) | 0.11 (0.83) | 1.10 (0.88) | 0.12 (0.83) |
| ^1^BOADICEA model with unaffected family members and PGS included, with no filter question | | | | | | | |

**Supplementary Table 4:** Comparison of referral processes, with each process compared directly to 10-year breast cancer status in the complete Generation Study cohort. Referral processes differ by inclusion/exclusion of unaffected family members (UFM) and polygenic scores (PGS), and filtering strategies.

| Model | Including unaffected family members | Including PGS | Filter Question*^1^* | Full Risk Assessment (%) (95% CI) | Referred (%)  (95% CI) | Sensitivity (%)*^2^* (95% CI) | Specificity (%)*^3^* (95% CI) | Precision (%)*^4^* (95% CI) | Number of risk assessments for one 10-year BC case to be referred (95% CI) |
| --- | --- | --- | --- | --- | --- | --- | --- | --- | --- |
| NICE | FALSE | FALSE | - | 11.0 (10.7, 11.3) | 1.7 (1.6, 1.8) | 5.0 (3.8, 6.3) | 98.4 (98.2, 98.5) | 5.2 (3.9, 6.6) | 123.8 (97.8, 164.9) |
| BOADICEA | TRUE | FALSE | 0 | 100.0 (100.0, 100.0) | 11.4 (11.1, 11.7) | 16.0 (13.8, 18.3) | 88.7 (88.4, 89.0) | 2.5 (2.1, 2.9) | 352.3 (306.2, 410.6) |
| BOADICEA | TRUE | FALSE | 1 | 11.0 (10.7, 11.3) | 7.7 (7.5, 7.9) | 13.7 (11.8, 15.8) | 92.4 (92.2, 92.6) | 3.2 (2.7, 3.7) | 45.1 (39.0, 52.9) |
| BOADICEA | TRUE | FALSE | 2 | 1.6 (1.5, 1.7) | 1.4 (1.3, 1.5) | 4.5 (3.3, 5.8) | 98.6 (98.5, 98.7) | 5.5 (4.1, 7.1) | 20.2 (15.8, 27.5) |
| BOADICEA | FALSE | FALSE | 0 | 100.0 (100.0, 100.0) | 13.8 (13.5, 14.1) | 18.2 (15.9, 20.6) | 86.3 (85.9, 86.6) | 2.3 (2.0, 2.7) | 309.1 (270.9, 357.3) |
| BOADICEA | FALSE | FALSE | 1 | 11.0 (10.7, 11.3) | 8.2 (7.9, 8.4) | 14.6 (12.6, 16.7) | 91.9 (91.7, 92.2) | 3.2 (2.7, 3.6) | 42.4 (36.9, 49.4) |
| BOADICEA | FALSE | FALSE | 2 | 1.6 (1.5, 1.7) | 1.5 (1.4, 1.6) | 4.5 (3.3, 5.8) | 98.5 (98.4, 98.6) | 5.3 (3.9, 6.8) | 20.2 (15.8, 27.5) |
| *^1^*Filter 0: All participants remain in referral process Filter 1: Participants without a family history of breast cancer removed from the referral process Filter 2: Participants without multiple family members with a history of breast cancer or one family member with breast cancer diagnosed before the age of 40 removed from the referral process | | | | | | | | | |
| *^2^*% of women who developed breast cancer within 10 years who were referred under that process | | | | | | | | | |
| *^3^*% of women who did not develop breast cancer withing 10 years who were not referred under that process | | | | | | | | | |
| *^4^*% of women who were referred under that process who developed breast cancer within 10 years | | | | | | | | | |

**Supplementary Table 5a:** Comparison of referral processes by NICE guidelines and different versions of the BOADICEA model to identify women estimated to be at above population level risk based on the most complete BOADICEA model, including unaffected family members (UFM) and polygenic scores (PGS) with no filter prior to risk assessment (filter 0). Comparison is within the subset of the study sample with non-missing age at menarche measurements.

| Model | Including Unaffected Family Members | Including PGS | Filter Question*^1^* | Full Risk Assessment (%) (95% CI) | Referred (%) (95% CI) | Sensitivity (%)*^2^* (95% CI) | Specificity (%)*^3^* (95% CI) | Precision (%)*^4^* (95% CI) | Number of risk assessments for one woman estimated to be at above population-level risk to be referred (95% CI) |  |
| --- | --- | --- | --- | --- | --- | --- | --- | --- | --- | --- |
| NICE | FALSE | FALSE | - | 10.8 (8.8, 13.0) | 1.4 (0.7, 2.3) | 4.8 (2.2, 7.9) | 99.8 (99.3, 100.0) | 88.4 (64.1, 99.9) | 8.7 (5.4, 18.7) |  |
| BOADICEA | TRUE | TRUE | 0 | 100.0 (100.0, 100.0) | 26.0 (22.4, 29.7) | Reference | Reference | Reference | 3.8 (3.4, 4.5) |  |
| BOADICEA | TRUE | TRUE | 1 | 10.8 (8.8, 13.0) | 6.3 (4.8, 8.0) | 24.3 (18.8, 30.7) | 100.0 (100.0, 100.0) | 100.0 (100.0, 100.0) | 1.7 (1.5, 2.1) |  |
| BOADICEA | TRUE | TRUE | 2 | 1.3 (0.6, 2.2) | 1.1 (0.5, 1.9) | 4.3 (1.8, 7.4) | 100.0 (100.0, 100.0) | 100.0 (100.0, 100.0) | 1.2 (1.0, 1.7) |  |
| BOADICEA | TRUE | FALSE | 0 | 100.0 (100.0, 100.0) | 11.9 (9.7, 14.5) | 29.3 (22.9, 36.6) | 94.2 (92.0, 96.0) | 64.1 (53.8, 73.7) | 13.1 (10.3, 17.2) |  |
| BOADICEA | TRUE | FALSE | 1 | 10.8 (8.8, 13.0) | 8.2 (6.5, 10.1) | 22.9 (17.5, 29.2) | 96.9 (95.5, 98.1) | 72.2 (61.7, 81.9) | 1.8 (1.5, 2.2) |  |
| BOADICEA | TRUE | FALSE | 2 | 1.3 (0.6, 2.2) | 1.3 (0.6, 2.2) | 4.3 (1.8, 7.4) | 99.7 (99.2, 100.0) | 84.9 (58.5, 99.8) | 1.2 (1.0, 1.7) |  |
| BOADICEA | FALSE | TRUE | 0 | 100.0 (100.0, 100.0) | 28.0 (24.3, 31.8) | 100.0 (100.0, 100.0) | 97.3 (95.8, 98.6) | 93.0 (89.0, 96.3) | 3.8 (3.4, 4.5) |  |
| BOADICEA | FALSE | TRUE | 1 | 10.8 (8.8, 13.0) | 6.5 (4.9, 8.2) | 24.3 (18.8, 30.7) | 99.8 (99.4, 100.0) | 97.8 (93.7, 100.0) | 1.7 (1.5, 2.1) |  |
| BOADICEA | FALSE | TRUE | 2 | 1.3 (0.6, 2.2) | 1.1 (0.5, 1.9) | 4.3 (1.8, 7.4) | 100.0 (100.0, 100.0) | 99.9 (99.4, 100.0) | 1.2 (1.0, 1.7) |  |
| BOADICEA | FALSE | FALSE | 0 | 100.0 (100.0, 100.0) | 14.2 (11.7, 16.9) | 31.6 (25.0, 39.0) | 92.0 (89.5, 94.2) | 58.2 (48.3, 67.6) | 12.1 (9.7, 15.8) |  |
| BOADICEA | FALSE | FALSE | 1 | 10.8 (8.8, 13.0) | 9.0 (7.2, 11.0) | 23.0 (17.6, 29.4) | 96.0 (94.3, 97.4) | 66.7 (56.1, 76.9) | 1.8 (1.5, 2.2) |  |
| BOADICEA | FALSE | FALSE | 2 | 1.3 (0.6, 2.2) | 1.3 (0.6, 2.2) | 4.3 (1.8, 7.4) | 99.7 (99.2, 100.0) | 84.9 (58.5, 99.8) | 1.2 (1.0, 1.7) |  |
| *^1^*Filter 0: All participants remain in referral process Filter 1: Participants without a family history of breast cancer removed from the referral process Filter 2: Participants without multiple family members with a history of breast cancer or one family member with breast cancer diagnosed before the age of 40 removed from the referral process | | | | | | | | | | |
| *^2^*% of women estimated to be above population-level risk based on the most complete BOADICEA model (reference) who were referred under that process | | | | | | | | | | |
| *^3^*% of women estimated to be at population-level risk based on the most complete BOADICEA model (reference) who were not referred under that process | | | | | | | | | | |
| *^4^*% of women who were referred under that process estimated to be above population-level risk based on the most complete BOADICEA model (reference) | | | | | | | | | | |

**Supplementary Table 5b:** Comparison of referral processes, with each process compared directly to 10-year breast cancer status in the subset of the study sample with non-missing age at menarche measurements. Referral processes differ by inclusion/exclusion of unaffected family members (UFM) and polygenic scores (PGS), and filtering strategies.

| Model | Including Unaffected Family Members | Including PGS | Filter Question*^1^* | Full Risk Assessment (%) (95% CI) | Referred (%) (95% CI) | Sensitivity (%)*^2^* (95% CI) | Specificity (%)*^3^* (95% CI) | Precision (%)*^4^* (95% CI) | Number of risk assessments for one 10-year BC case to be referred (95% CI) |
| --- | --- | --- | --- | --- | --- | --- | --- | --- | --- |
| NICE | FALSE | FALSE | - | 10.8 (8.8, 13.0) | 1.4 (0.7, 2.3) | 4.5 (2.3, 7.4) | 98.7 (97.7, 99.4) | 5.2 (2.3, 12.6) | 147.9 (87.2, 302.6) |
| BOADICEA | TRUE | TRUE | 0 | 100.0 (100.0, 100.0) | 26.0 (22.4, 29.7) | 37.2 (29.6, 44.9) | 74.2 (70.4, 77.8) | 2.3 (1.7, 3.0) | 166.9 (131.0, 220.5) |
| BOADICEA | TRUE | TRUE | 1 | 10.8 (8.8, 13.0) | 6.3 (4.8, 8.0) | 16.6 (11.2, 22.7) | 93.8 (92.1, 95.4) | 4.2 (2.6, 6.4) | 40.4 (27.4, 64.0) |
| BOADICEA | TRUE | TRUE | 2 | 1.3 (0.6, 2.2) | 1.1 (0.5, 1.9) | 3.9 (1.8, 6.6) | 98.9 (98.1, 99.6) | 5.6 (2.2, 15.3) | 21.1 (8.3, 53.1) |
| BOADICEA | TRUE | FALSE | 0 | 100.0 (100.0, 100.0) | 11.9 (9.6, 14.5) | 19.4 (13.3, 26.1) | 88.2 (85.6, 90.6) | 2.6 (1.7, 3.9) | 320.1 (228.4, 485.2) |
| BOADICEA | TRUE | FALSE | 1 | 10.8 (8.8, 13.0) | 8.2 (6.5, 10.2) | 16.5 (11.1, 22.7) | 91.9 (89.9, 93.7) | 3.2 (2.0, 4.8) | 40.6 (27.5, 64.3) |
| BOADICEA | TRUE | FALSE | 2 | 1.3 (0.6, 2.2) | 1.3 (0.6, 2.2) | 4.2 (2.0, 6.9) | 98.7 (97.8, 99.5) | 5.1 (2.1, 12.8) | 19.7 (7.8, 46.9) |
| BOADICEA | FALSE | TRUE | 0 | 100.0 (100.0, 100.0) | 28.0 (24.3, 31.7) | 39.7 (32.0, 47.5) | 72.2 (68.5, 75.9) | 2.3 (1.7, 3.0) | 156.2 (123.4, 203.9) |
| BOADICEA | FALSE | TRUE | 1 | 10.8 (8.8, 13.0) | 6.5 (4.9, 8.2) | 16.8 (11.3, 22.9) | 93.7 (92.0, 95.3) | 4.2 (2.6, 6.3) | 40.0 (27.2, 62.8) |
| BOADICEA | FALSE | TRUE | 2 | 1.3 (0.6, 2.2) | 1.1 (0.5, 1.9) | 4.0 (1.9, 6.7) | 98.9 (98.1, 99.6) | 5.7 (2.3, 15.6) | 20.6 (8.1, 50.7) |
| BOADICEA | FALSE | FALSE | 0 | 100.0 (100.0, 100.0) | 14.2 (11.6, 16.8) | 20.3 (14.2, 27.1) | 85.9 (83.2, 88.5) | 2.3 (1.5, 3.3) | 305.3 (219.7, 451.4) |
| BOADICEA | FALSE | FALSE | 1 | 10.8 (8.8, 13.0) | 9.0 (7.1, 11.0) | 17.1 (11.6, 23.4) | 91.1 (89.1, 93.0) | 3.1 (2.0, 4.6) | 39.2 (26.7, 61.1) |
| BOADICEA | FALSE | FALSE | 2 | 1.3 (0.6, 2.2) | 1.3 (0.6, 2.2) | 4.2 (2.0, 6.9) | 98.7 (97.8, 99.5) | 5.1 (2.1, 12.8) | 19.7 (7.8, 46.9) |
| *^1^*Filter 0: All participants remain in referral process Filter 1: Participants without a family history of breast cancer removed from the referral process Filter 2: Participants without multiple family members with a history of breast cancer or one family member with breast cancer diagnosed before the age of 40 removed from the referral process | | | | | | | | | |
| *^2^*% of women who developed breast cancer within 10 years who were referred under that process | | | | | | | | | |
| *^3^*% of women who did not develop breast cancer withing 10 years who were not referred under that process | | | | | | | | | |
| *^4^*% of women who were referred under that process who developed breast cancer within 10 years | | | | | | | | | |

**Supplementary Figure 1:** Receiver operating characteristic (ROC) graph for 4 complexities of BOADICEA, predicting 10-year absolute risk of breast cancer.

**Supplementary Figure 2:** Calibration plots for 4 complexities of BOADICEA, predicting 10-year absolute risk of breast cancer. For each model, weighted study population is split into deciles of absolute risk and the expected and observed risk per decile is plotted with 95% confidence intervals for observed risk. Study population size: 1,256 with 392 10-year invasive/DCIS breast cancer cases.

**Supplementary Figure 3:** Venn diagram showing which components of the BOADICEA referral process (lifetime risk, risk between ages 40 and 50 (40-50 Risk), and BRCA risk) trigger referral in the most complete BOADCIEA referral process (No filter, including unaffected family members and PGS)

**Supplementary Figure 4:** Venn diagram showing the comparisons of proportions of the weighted study sample referred by three methods: NICE criteria, BOADICEA with no filter including PRS (BOADICEA + PGS), and BOADICEA with no filter and including PGS and unaffected family members (UFM) (BOADICEA + PRS + UFM)
